# Reversible mechano-electrochemical writing of metallic nanostructures with the tip of an atomic force microscope

**DOI:** 10.3762/bjnano.3.92

**Published:** 2012-12-05

**Authors:** Christian Obermair, Marina Kress, Andreas Wagner, Thomas Schimmel

**Affiliations:** 1 Institute of Applied Physics and Center for Functional Nanostructures (CFN), Karlsruhe Institute of Technology (KIT), 76128 Karlsruhe, Germany,; 2 Institute of Nanotechnology (INT), Karlsruhe Institute of Technology (KIT), 76021 Karlsruhe, Germany

**Keywords:** atomic force microscopy, electrochemical deposition, electrochemistry, nanoelectronics, nanofabrication, nanolithography, nanotechnology, MEMS and NEMS, reversible processes, scanning probe microscopy and lithography

## Abstract

We recently introduced a method that allows the controlled deposition of nanoscale metallic patterns at defined locations using the tip of an atomic force microscope (AFM) as a “mechano-electrochemical pen”, locally activating a passivated substrate surface for site-selective electrochemical deposition. Here, we demonstrate the reversibility of this process and study the long-term stability of the resulting metallic structures. The remarkable stability for more than 1.5 years under ambient air without any observable changes can be attributed to self-passivation. After AFM-activated electrochemical deposition of copper nanostructures on a polycrystalline gold film and subsequent AFM imaging, the copper nanostructures could be dissolved by reversing the electrochemical potential. Subsequent AFM-tip-activated deposition of different copper nanostructures at the same location where the previous structures were deleted, shows that there is no observable memory effect, i.e., no effect of the previous writing process on the subsequent writing process. Thus, the four processes required for reversible information storage, “write”, “read”, “delete” and “re-write”, were successfully demonstrated on the nanometer scale.

## Introduction

The electrochemical deposition of metallic contacts, wires and patterns on the nanometer scale is of tremendous technological relevance for contacting micro- and nanoelectronic integrated circuits in the semiconductor industry [1]. With the shrinking dimensions of semiconductor structures, there is a need to understand and control electrochemical deposition processes on the nanometer scale. This also applies to the field of micro- and nano-electromechanical systems (MEMS and NEMS). At the same time, much progress was achieved in recent years in understanding the mechanisms and developing new methods for the deposition and control of metallic structures and contacts on the nanometer scale and even down to the atomic scale [2–13]. Nanowires showing a dramatic increase of the yield strength by more than one order of magnitude have been fabricated by electrochemical deposition [14]; transport through single molecules between metallic nanocontacts was studied intensively [15–24]. Even a single-atom transistor [25–29] was demonstrated, i.e., a device that allows the controlled switching “on” and “off” of an electrical current by the controlled and reversible movement of one individual atom.

While electrochemical processes on the nanometer scale are of increasing technological relevance, there is also a need to visualize, study and control these processes locally on this length scale. Atomic force microscopy (AFM) and related techniques are valuable tools for imaging surfaces and surface processes on the nanometer scale. Examples of recent work can be found in the literature [30–40]. At the same time, the scanning tips of the atomic force microscope, the scanning tunneling microscope (STM) and related instruments can be used as tools for surface modification and nanolithography [9–12,41–48], even down to the atomic scale [41–45]. The great advantage of these instruments for nanoelectrochemistry is the fact that they also allow the in situ and real-time observation of the processes within the electrochemical cell [49–53], making them a valuable tool for studying electrochemical processes on the nanometer scale.

Recently, we demonstrated the local electrochemical deposition of metallic nanostructures and nanowires mechanically induced with the tip of an AFM [12]. In the semiconductor industry, mechano-electrochemical processes are frequently used in the form of mechano-electrochemical polishing. While this is only a macroscopic method with very poor lateral resolution, the tip of an AFM allows the site-selective control of electrochemical deposition on the nanometer scale, e.g., by mechanically depassivating a surface. Here, we study this process in more detail. We demonstrate that the technique of AFM-tip-induced mechano-electrochemical writing is fully reversible, and we investigate the long-term stability of the resulting structures. We also discuss the implications of these results on the understanding of the deposition mechanism.

## Results and Discussion

### Reversible writing, deleting and re-writing

**Figure 1:**
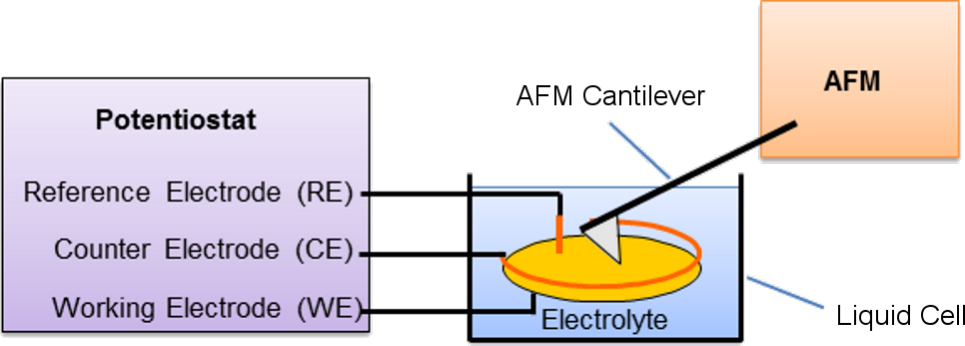
Schematic diagram of an electrochemical atomic force microscope, comprising an atomic force microscope with a laser-beam detection system (not shown), an electrochemical liquid cell with reference electrode (RE), counter electrode (CE) and working electrode (WE), and a computer-controlled potentiostat. The cantilever is dipped into the electrolyte but not directly connected to the electrodes.

Writing of metallic structures with the tip of an atomic force microscope as a “nano-electrochemical pen” was performed as described in our previous work [12]. A glass substrate covered with a polycrystalline gold film is used as a substrate. When placed into an electrolyte solution containing copper sulfate and H_2_SO_4_, a thin copper oxide containing film is formed on the gold surface, its thickness and properties depending on the pH value of the solution. This film can act as a passivation layer preventing electrochemical deposition of copper when an electrochemical deposition potential is applied to the gold electrode. Our tip-activated deposition method now works in the following way: while an electrochemical deposition voltage is applied to the gold electrode, the AFM tip repeatedly scans along a certain pathway at force loads on the order of 10 nN, in this way locally destroying or damaging the oxide-based passivation layer on the gold electrode. As a consequence, copper is deposited on the gold electrode at exactly the locations where the tip has destroyed the passivation layer, i.e., along the lines scanned by the tip when the deposition voltage is applied. After this site-selective, tip-activated deposition, the deposition potential is switched off and an electrochemical potential close to the electrochemical equilibrium is applied, i.e., no further deposition and no further dissolution occurs. Subsequently, AFM contact-mode imaging of the resulting structures is performed, i.e., nanopatterning and AFM imaging are performed with one and the same tip in situ within the electrolyte. This process has the advantage that an entire sequence of deposition and dissolution processes can be performed, and after each step, the resulting structures and their changes can be visualized and quantitatively characterized by AFM. A schematic representation of the experimental setup is given in Figure 1.

In order to gain further insights into the mechanism of tip-induced electrochemical deposition, the erasability of the deposited structures and the reversibility of the deposition process were investigated in more detail. Therefore, a copper structure was deposited electrochemically by local mechano-electrochemical AFM-tip activation. Subsequently the copper structure was dissolved by applying an electrochemical dissolution potential. In a following step a new and differently shaped copper nanostructure was deposited at the same location on the sample.

**Figure 2:**
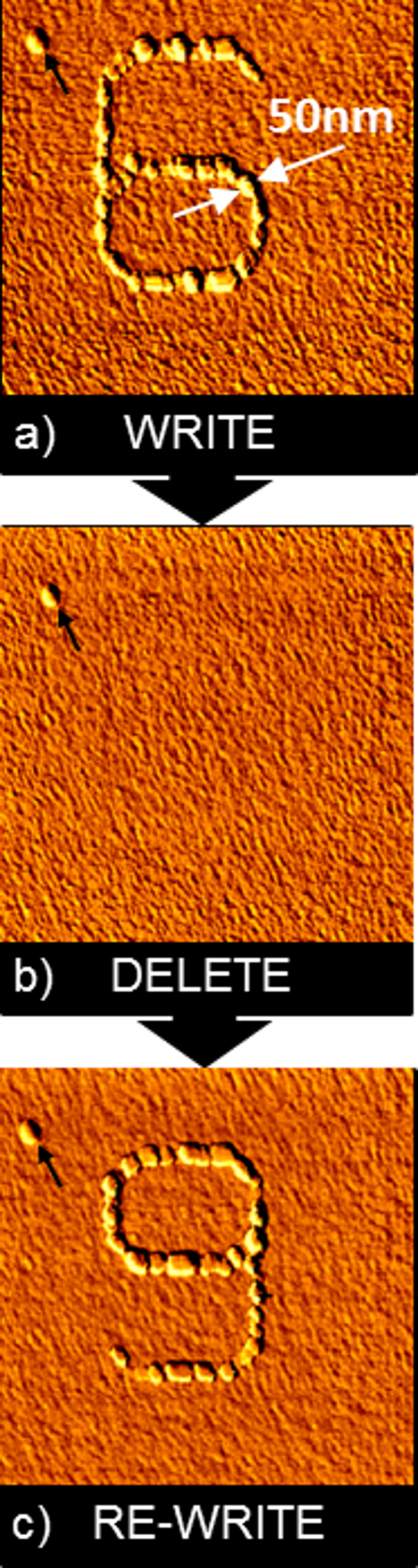
Reversible mechano-electrochemical writing of Cu nanostructures. (a) WRITE: In situ AFM image of an initial Cu island structure (“6”). The “6”-structure was deposited by tip-induced mechano-electrochemical writing on a gold substrate, applying a deposition potential of −60 mV versus Cu/Cu^2+^ for 5 s. (b) DELETE: In situ AFM image of the gold substrate after deletion. For deletion a potential of +0.2 mV versus Cu/Cu^2+^ was applied for 180 s. (c) RE-WRITE: In situ AFM image of a further Cu island structure (“9”). The “9”-structure was deposited on the same area where previously the “6”-structure had been written and deleted. For orientation see the point-shaped defect in the upper-left corner of the images, marked by an arrow. Typical height of the islands: 15 nm. Scan size: 1.6 µm × 1.6 µm.

In Figure 2 the result of such a deposition experiment is given. Figure 2 shows the AFM topography of one and the same 1.6 µm × 1.6 µm scanning area of a polycrystalline gold surface in an electrolyte containing Cu^2+^ ions. The electrolyte consisted of an aqueous solution of 50 mM H_2_SO_4_ (suprapure, Merck) with 1 mM CuSO_4_ (p.a., Merck). All potentials were measured against a Cu/Cu^2+^ reference electrode, using a Cu counter electrode and the polycrystalline gold surface as the working electrode.

For the experiment, it was important to make sure that after the first Cu deposition, after dissolution of this Cu nanostructure and deposition of a new Cu structure on the gold surface, always exactly the same area was imaged with the AFM. For this reason, an area was selected for the experiment which contained an easily identifiable label within the scanning area in the form of a gold-coated hillock on the otherwise smooth gold film (see arrow in the AFM images of Figure 2a–c). This gold-coated hillock-like defect was not dissolved, even at a high underpotential of +1 V, which guaranteed that it remained unchanged at the potentials applied during the copper deposition and dissolution experiments.

In the first step, a deposition potential of −60 mV was applied and the gold surface was scanned with the AFM tip following the contour of the shape of the digit “6” with 13 repetitions within 5 s. The scanning rate of the AFM tip on the sample surface was about 8 µm/s. The force load of the AFM tip was approx. 10 nN. In this way a line structure of largely interconnected copper islands with the shape of the digit “6” was deposited. Subsequently a potential of −30 mV (the “holding potential” at which no further Cu deposition took place) was applied to the gold working electrode and the surface area was then scanned with the AFM.

The contact-mode AFM image of Figure 2a shows that the deposition process is highly selective, i.e., the deposition occurred at exactly the sites scanned by the AFM tip, whereas absolutely no copper islands were deposited at other places. The metal deposition starts in the form of small islands, most probably nucleating at atomic-scale defects and grain boundaries. The deposited metal in the beginning of the deposition process thus forms a chain of islands along the path of the AFM tip. These islands finally touch and overgrow each other, in this way forming a continuous wire in the shape of the digit “6”. The line width of the structure is approx. 50 nm. The force load of the AFM tip during imaging was the same as during the deposition process (approx. 10 nN).

In the second step a dissolution potential of +0.2 V was applied for 180 s to the Au working electrode. Figure 2b shows an AFM image of the same scanning area as shown in Figure 2a, but after application of the dissolution potential for 180 s. All copper islands are dissolved. During the dissolution process, the AFM tip was still in contact with the sample surface, but it was not scanning, i.e., it was not moved at all in relation to the sample surface. In Figure 2b no tip-induced surface defects are found on the sample surface.

Following the same procedure described above for the deposition of the copper structure with the shape of the digit “6”, a new structure with the shape of the digit “9” was deposited after dissolution of the “6”, at exactly the same position of the sample. During the deposition time of approx. 5 s the deposition potential of −60 mV was applied. During this time, the AFM tip was continuously scanning along the trace of the figure “9”. In total, this scan was repeated 13 times within this deposition time. This means that the repetition rate with which the tip reactivated the same position of the sample during deposition was once every 0.4 seconds. The result of subsequent AFM imaging, again at a “holding” potential of −30 mV, is given in Figure 2c. In the same scanning area, where previously copper islands with the shape of the digit “6” were deposited and subsequently dissolved, new cooper islands with the shape of the digit “9” were deposited. Again, the deposition process was highly selective, i.e., no copper islands were found in the scanned area, except those arranged in the shape of the digit “9”.

Neither in Figure 2b nor in Figure 2c can any indications of the previous deposition processes be found. This leads to the conclusion that during AFM-tip-induced electrochemical deposition with force loads of about 10 nN, as used in the previous experiment, the gold surface itself was not modified. Obviously, only the passivation layer on top of the gold surface was locally removed by means of the AFM tip scanning along the path of the digits “6” and “9”. During none of our electrochemical deposition experiments was any observable tip-induced surface damage or defect found.

This allows performing sequential electrochemical deposition on one and the same area of the sample, because no modification of the sample area remains after the dissolution of the previously deposited structures. The tip-induced electrochemical deposition of copper islands onto gold surfaces is fully reversible.

### Self-passivation and long-time stability

A key feature for the technological relevance and application perspectives of electrochemically deposited nanostructures is their long-term stability. The nanostructures have to remain stable under ambient air after being taken out of the electrolyte.

To study the long-term stability of electrochemically deposited nanostructures written with the AFM tip as a mechano-electrochemical pen, a copper structure was deposited following the procedure described above. To make the finding of the same structure after longer periods of time as well as its study with scanning electron microscopy (SEM) easier, a larger structure was deposited by AFM with a total size of 6 µm × 6 µm. The copper structure with the shape of a square frame was again deposited by AFM tip-induced electrochemical deposition on a polycrystalline gold substrate. During deposition, the structure written in the shape of a square-shaped spiral with an inner-edge length of 2 µm and an outer-edge length of 6 µm was scanned 45 times with the tip of the AFM. The AFM path during the structuring process consisted of 15 concentric squares becoming subsequently smaller in size starting at a length of 6 µm and ending at a length of 2 µm. The structures were written by using a tip speed relative to the sample surface of approx. 50 µm/s, resulting in a repetition rate of 5 seconds with which the tip subsequently retraced the same profile, i.e., the same position along the scanning path of the tip was hit by the tip every 5 seconds. The whole writing cycle was repeated 45 times, resulting in a total writing time of 3.7 minutes. The force load of the AFM tip again was approx. 10 nN. During deposition, a deposition potential of −70 mV versus Cu/Cu^2+^ was applied to the gold electrode. An aqueous solution of 100 mM H_2_SO_4_ (suprapure, Merck) with 1 mM CuSO_4_ (p.a., Merck) was used as electrolyte. After deposition, a holding potential of −20 mV versus Cu/Cu^2+^ was applied to the gold electrode.

After deposition, the AFM tip was taken out of the liquid cell and the electrolyte was poured out of the cell as quickly as possible to avoid further deposition and dissolution during this very short transitional phase between the sample being immersed in the electrolyte and the sample being dry in ambient air, i.e., a transitional phase with an undefined electrochemical potential. During the pouring out of the electrolyte, the holding potential was kept applied to the electrodes in order to maintain defined electrochemical conditions as long as possible. Subsequently, the gold substrate with the copper structure was rinsed with bi-distilled water and then dried at room temperature.

**Figure 3:**
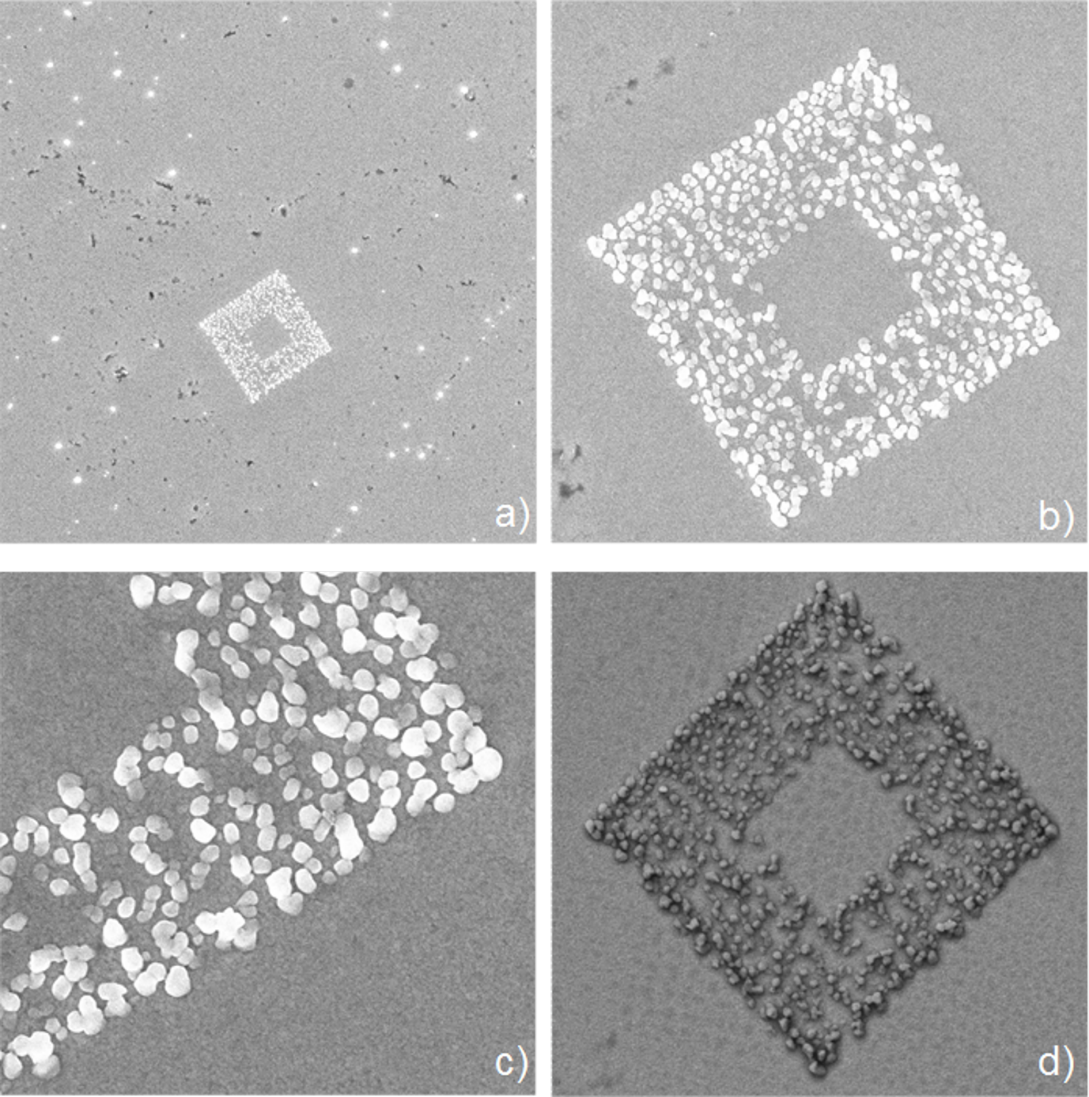
SEM image demonstrating the ex situ stability of the tip-induced electrochemically deposited copper islands on a gold substrate. (a–c) SEM image immediately taken after deposition, (d) SEM image of the same sample area after 1.5 years. Scan size: (a) 32 µm × 32 µm, (b) 9 µm × 9 µm, (c) 4 µm × 4 µm, (d) 9 µm × 9 µm.

Figure 3a–c shows SEM images of the as-prepared copper structure one day after deposition. The SEM images were taken with a Zeiss SEM equipped with a field-emission electron source. In Figure 3a a 32 µm × 32 µm scanning area of the sample is given. In the middle of the scanning area, the square structure of copper islands with an open area in the center is visible. Figure 3b and Figure 3c are zoomed SEM images of the same structure.

To demonstrate the long-term stability of the electrochemically deposited copper structure, the same sample area of Figure 3b was investigated again after more than one year. Figure 3d shows an SEM image of the same 9 µm × 9 µm scanning area as in Figure 3b 1.5 years after the electrochemical deposition of the copper structure, thus showing the long-term stability of the resulting structures under ambient conditions. The typical humidity of the laboratory air over this time period of 1.5 years varied between 30% and 90% relative humidity, for most of the time being between 40% and 60%.

The different imaging contrasts between Figure 3a–c and Figure 3d are due to the use of different SEM detectors. For Figure 3a–c an in-lens SEM detector was used. For the image of Figure 3d the signal from a secondary electron detector was used leading to dominant topographic information.

The copper structure in Figure 3d remains unchanged, compared to Figure 3b although the sample was stored for 1.5 years under ambient conditions, i.e., ambient air and ambient humidity. If two different detectors for the SEM imaging had not been used, the structures would not have shown any discernible change at all during this 1.5-year period of time.

### Nucleation density, pattern homogeneity and island size

When comparing the structures of Figure 2 with the structures of Figure 3, striking differences are observed:

- In both cases, the structures consist of a sequence of copper islands. In the case of Figure 2, these islands are significantly smaller (approximate diameter: 50 nm) than in Figure 3 (typically 80–200 nm).
- In the case of Figure 2 the islands are largely interconnected to form continuous lines (“nanowires”), whereas in Figure 3 they are mostly found in the form of single, isolated islands or in the form of smaller aggregates of 2–5 islands interconnected with each other, but separated from the rest of the copper islands.
- In the case of Figure 2, the islands form a very regular sequence and have a much narrower size distribution. In Figure 3, the positions of the islands as well as their local areal density strongly fluctuate, which is also true for the island sizes.

These differences are remarkable, considering that the structures of Figure 2 and Figure 3 have been deposited with the same technique and by applying similar electrochemical parameters such as concentrations and pH of the electrolyte, deposition potential, and holding potential. The major difference was in the very different experimental parameters for the repetition rate of the AFM scanning process during structure growth, which was by a factor of more than 12 lower for the pattern of Figure 3 than for the structure of Figure 2. This, in turn, allows conclusions to be drawn concerning the structuring mechanism.

The results described above can be explained in the following way: obviously, in both cases, there is a growth of individual copper islands along the lines where the passivating layer is damaged or removed with the tip of the AFM. The results of previously reported [12] already showed that once an area or line along the surface is activated with the AFM tip, the passivation layer starts to regrow within a certain time, which was typically on the order of seconds (depending on the chemical parameters of the system such as concentrations, pH value), requiring a continuous reactivation of the active area of the surface with the tip of the AFM to allow for the nucleation of further islands. Assuming this, a repeated scanning of the same line or trace with the AFM tip is needed during the structuring process to prevent repassivation of the tip-activated areas before the deposition process is completed. For this purpose, obviously the repetition rate of tip-induced reactivation has to be higher than the inverse time constant of the self-passivation process of the surface.

Thus the low density of islands in the case of Figure 3 is easily explained by the much lower rate of reactivation of the surface along the scan trace with the AFM tip (only every 5 seconds), allowing a repassivation between consecutive AFM scans, resulting in the growth of fewer, larger islands. In the case of Figure 2, the reactivation took place every 0.4 seconds, allowing the formation of a continuous chain of much smaller islands and showing no effects of repassivation in the form of parts of the line in which no islands grew. This indicates a repassivation time in between the repetition times of these two experiments, in agreement with values in the range of seconds estimated previously [12].

## Conclusion

The controlled and reversible electrochemical writing of copper nanostructures on a polycrystalline gold surface was achieved with the tip of an atomic force microscope used as a mechano-electrochemical pen. After dissolution of a certain structure by reversing the electrochemical potential, new, different structures were written at the same position with no observable memory effect of the previous writing process. This demonstrates that the tip-induced modification is limited to the passivation layer and does not change the topography or the properties of the gold substrate, and it supports the proposed mechanism of site-selective, local AFM-tip-induced depassivation of the surface. Finally, it is shown that a sufficiently high repetition rate of the AFM scan during the growth process is needed to prevent repassivation of the once-activated surface area during the growth process, and an estimate of the repassivation time constant is derived from the experimental results.

To conclude, the four basic processes required for reversible information storage, i.e., “Write”, “Read”, “Delete” and “Re-write”, were successfully demonstrated on the nanometer scale. Long-term stability of the resulting structures under ambient air for more than 1.5 years was demonstrated, which can be explained by self-passivation of the surface.

Apart from the aspect of reversible nanolithography, the results also help in understanding microscopic mechanisms of mechanically activated or mechanically assisted electrochemical processes on metallic surfaces, e.g., during electropolishing or in combined mechanical wear and corrosion processes.

## Experimental

The experimental setup was described previously [12] in more detail. An AFM, constructed at our institute, that was specially equipped for electrochemical nanolithography was used. An electrochemical cell (diameter: approx. 20 mm), equipped with Cu reference and counter electrodes (consisting of copper wires of 0.5 mm diameter from Goodfellow) were used. Glass substrates with evaporated gold films were used as working electrodes, on which the deposition experiments were performed. Rinsing, sonication and gold coating of the glass substrates was performed following the procedure described previously [12]. Deposition and dissolution was directed by a computer-controlled bi-potentiostat.

All AFM images were taken in situ within the electrolyte in contact mode and represent original data that are shown without filtering. V-shaped silicon nitride cantilevers were used with force constants between 0.03 and 0.1 N/m. Cyclic voltammograms were measured both before and after each experiment.
